# Ligand-Based Virtual Screening, Molecular Docking, Molecular Dynamics, and MM-PBSA Calculations towards the Identification of Potential Novel Ricin Inhibitors

**DOI:** 10.3390/toxins12120746

**Published:** 2020-11-26

**Authors:** Fernanda D. Botelho, Marcelo C. dos Santos, Arlan da S. Gonçalves, Kamil Kuca, Martin Valis, Steven R. LaPlante, Tanos C. C. França, Joyce S. F. D. de Almeida

**Affiliations:** 1 Laboratory of Molecular Modeling Applied to Chemical and Biological Defense, Military Institute of Engineering, Praca General Tiburcio 80, Rio de Janeiro 22290-270, Brazil; fernanda.botelho@ime.eb.br (F.D.B.); marcelocarneiro.mcs@gmail.com (M.C.d.S.); 2 Federal Institute of Education, Science and Technology, Avenida Ministro Salgado Filho, 1000, Vila Velha 29106-010, Brazil; arlansgoncalves@gmail.com; 3 Department of Chemistry, Faculty of Science, University of Hradec Kralove, Rokitanskeho 62, 50003 Hradec Kralové, Czech Republic; 4 Department of Neurology of the Medical Faculty of Charles University and University Hospital in Hradec Kralove, Sokolska 581, 50005 Hradec Kralove, Czech Republic; martin.valis@fnhk.cz; 5 INRS—Institut Armand-Frapier, 531 Boulevard des Prairies, Laval, QC H7V 1B7, Canada; Steven.LaPlante@inrs.ca

**Keywords:** ricin, ricin inhibitors, molecular dynamics, ligand-based virtual screening, chemical/biological warfare agents

## Abstract

Ricin is a toxin found in the castor seeds and listed as a chemical weapon by the Chemical Weapons Convention (CWC) due to its high toxicity combined with the easiness of obtention and lack of available antidotes. The relatively frequent episodes of usage or attempting to use ricin in terrorist attacks reinforce the urge to develop an antidote for this toxin. In this sense, we selected in this work the current RTA (ricin catalytic subunit) inhibitor with the best experimental performance, as a reference molecule for virtual screening in the PubChem database. The selected molecules were then evaluated through docking studies, followed by drug-likeness investigation, molecular dynamics simulations and Molecular Mechanics Poisson–Boltzmann Surface Area (MM-PBSA) calculations. In every step, the selection of molecules was mainly based on their ability to occupy both the active and secondary sites of RTA, which are located right next to each other, but are not simultaneously occupied by the current RTA inhibitors. Results show that the three PubChem compounds 18309602, 18498053, and 136023163 presented better overall results than the reference molecule itself, showing up as new hits for the RTA inhibition, and encouraging further experimental evaluation.

## 1. Introduction

Ricin is a toxin found in the seeds of the castor plant (*Ricinus communis*), a widely spread plant in tropical regions and against which there are still no antidotes. It is classified as a ribosome-inactivating protein (RIP) due to its depurination role in the eukaryotic cells, which consist of the removal of a single adenine located in the universally conserved GAGA-tetraloop structure of 28S ribosomal RNA (rRNA) of eukaryotic cells. The removed adenine is the second nitrogenous base in the referred tetraloop, which is underlined, and corresponds to adenine 4324 (A-4324) of rat 28S rRNA [1,2]. Since rRNA is involved in protein synthesis and elongation, the cleavage of the glycosidic bond and consequent removal of the adenine interrupts these processes and potentially leads to cell death [1,3].

Ricin is classified as a type 2 RIP since it is formed by two subunits, ricin toxin A (RTA) and ricin toxin B (RTB), linked by a disulfide bridge between Cys259 of RTA and Cys4 of RTB [4,5]. RTB is a lectin that promotes cell entrance due to interaction with galactose-containing glycolipids and glycoproteins in the cell membrane. Once inside the cell, ricin is subjected to retrograde transport towards the endoplasmic reticulum, where RTA and RTB are separated by the enzyme disulfide isomerase [4,5]. Then, RTA, the catalytic subunit, moves to the cytosol where it encounters the rRNA and removes the aforementioned adenine, in a process called depurination. This process is very fast: RTA can inactivate up to 1500 ribosomes per minute [2,6], explaining why ricin is so toxic. Although it is difficult to define a number of castor seeds that can potentially kill a human due to plant variations, and it is estimated that the ingestion of 8–20 castor seeds can be lethal for adults. Additionally, ingestion is not the only route through which ricin can penetrate the human body. This can also happen through inhalation, which is the most toxic way of exposure, and injection that is also an effective route of intoxication by ricin [3,7].

Ricin is listed as a chemical weapon by the chemical Weapons Convention (CWC) [8] since it is not only highly toxic, but also easy to obtain and soluble in water, characteristics that increase its potential of being used in terrorist attacks. Many episodes of ricin usage were registered through the years and the most well-known is the murder of the Bulgarian dissident Georgi Markov in 1978, in London, in the so-called “umbrella murder” [9]. More recently, in September 2020, a letter addressed to the White House was intercepted and the analysis confirmed the presence of ricin [10]. Additionally, the castor oil, extracted from the castor plant, is a lubricant with many applications in the chemical industry, so the accidental intoxication of workers involved in the oil extraction must also be considered. Animal intoxication is also possible since the castor plant is naturally found in countries such as Brazil, China, and India [11]. For all these reasons, the search for an antidote against ricin intoxication must continue.

The catalytic mechanism of RTA is well-known, and the residues involved in catalytic activity or in substrate complexation were already identified. The catalytic residues, i.e., the ones directly involved in breaking the adenine glycosidic bond, are Glu177 and Arg180. Other residues located in the active site are responsible for the RTA-substrate complex stabilization, which are Tyr80, Val81, Gly121, Tyr123, Asn209, and Trp211. In addition, it was observed that the guanine located right before the adenine that is removed (corresponding to guanine 4323—G-4323—of rat rRNA), accommodates inside a secondary pocket in RTA. In this secondary pocket, guanine forms hydrogen bonds (H-bonds) with Asp75, Asn78, Asp96, and Asp100 [12,13].

The competitive RTA inhibitors currently reported in literature are able to inhibit it only at the micromolar range [14,15,16]. This suggests that there is still room for the search of compounds capable of achieving the nanomolar range necessary for an effective inhibition and consequent neutralization of the RTA action inside the cell. To move on this direction, we started with the molecular structure of the competitive inhibitor N-(N-(pterin-7-yl)carbonylglycyl)-L-tyrosine, called NNPT here, which showed the smallest IC_50_ reported so far against ricin (6 µM) [15]. Through the application of the ligand-based virtual screening (LBVS) technique on the PubChem database (https://pubchem.ncbi.nlm.nih.gov/) [17], we found molecules similar to NNPT and evaluated them through molecular docking, molecular dynamics (MD) simulations, and Molecular mechanics—Poisson–Boltzmann Surface Area (MM-PBSA) calculations, in order to verify their behavior when bound to RTA. Similar computational methodologies have been applied before and proven to lead to promising experimental results [18,19,20,21]. We postulated that a ligand that is capable of simultaneously stablishing H-bonds with residues of the active site and of the secondary site of RTA is more likely to show satisfactory inhibitory activity than the current inhibitors that were already shown to occupy solely the active site [14,15,16]. Thus, our ligand selection was primarily based on the analysis of H-bonds formed between the ligand and residues of the active and secondary sites of RTA. NNPT was also submitted to docking and MD simulations in order to provide reference parameters for comparison since it is known to show inhibitory activity against RTA in experimental tests.

## 2. Results

### 2.1. Protein Preparation and Redocking Procedure

**Figure 1:**
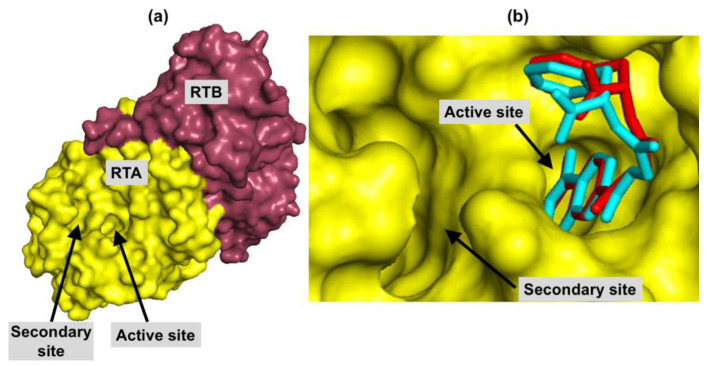
(**a**) localization of RTA active and secondary sites with respect to ricin complete structure. RTA surface is in yellow and RTB surface is in pink (PDB code: 3RTI); (**b**) best ranked pose, in red, and experimental position, in cyan of NNPCP inside RTA, whose surface is yellow. All ligand hydrogens are hidden for better clarity.

The spherical search space, defined to comprise both active and secondary binding pockets of RTA, had center coordinates at *x* = 8.64; *y* = −24.68 and *z* = −8.78 and a radius of 10.0 Å. Figure 1a indicates ricin complete structure (Protein Data Bank—PDB—code: 3RTI; the crystallized ligand was removed for figure construction) and the location of these two binding pockets.

The redocking procedure performed in the Molegro Virtual Docker (MVD^®^) software [22], resulted in a root-mean-square deviation (RMSD) of 0.77 Å between the best ranked pose and the experimental position of the ligand N-(N-(pterin-7-yl)carbonylglycyl)-L-phenylalanine (called here NNPCP) inside the PDB (https://www.rcsb.org/) structure 4HUO of RTA (Figure 1b). Since this RMSD value is smaller than 2.0 Å, the docking procedure was considered valid according to the literature recommendation [23]. As expected, the co-crystallized ligand is located inside the active site since it is a competitive inhibitor; and it is clear that the secondary site is empty. The absence of interactions on this site may explain the relatively high IC_50_ value of 20 µM observed for this ligand [15].

### 2.2. LBVS, Ligand Preparation, and Target Prediction

**Figure 2:**
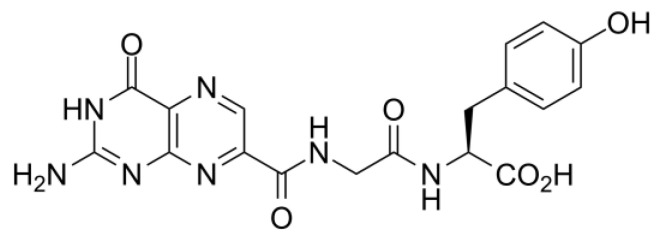
Structure of N-(N-(pterin-7-yl)carbonylglycyl)-L-tyrosine (NNPT).

The competitive inhibitor used as the reference compound for LBVS is NNPT, which showed an IC_50_ of 6 µM [15] (Figure 2).

The search for molecules that are at least 80% similar to NNPT at PubChem data base https://pubchem.ncbi.nlm.nih.gov/) [17] resulted in 1252 Simplified Molecular-Input Line-Entry System (SMILES) codes. After submission of all those SMILES codes to LigPrep for 3D structure generation and optimization, a set containing 2528 molecules was obtained. The expansion of the molecules set occurred due to the generation of estereoisomers and protonated/deprotonated species at pH 7.4.

The ligands were submitted to the HitPick web server (https://mips.helmholtz-muenchen.de/hitpick/cgi-bin/index.cgi?content=targetPrediction.html) for target prediction and the results are shown in Table S1. Since HitPick deals with SMILES codes as inputs, the original set containing the 1252 SMILES codes was submitted to this web server for target prediction. Nearly 100 molecules presented prediction precision greater than 80%, indicating that only ~8% of the molecules has a relatively high probability of binding other proteins and not RTA. Thus, none of the molecules was eliminated from the original set.

### 2.3. Molecular Docking

Docking results using the Protein-Ligand ANT System (PLANTS) docking algorithm at the Cheminformatic Tools and Databases for Pharmacology (Chemoinfo) (https://chemoinfo.ipmc.cnrs.fr/) [24,25] to evaluate all 2528 molecules were analyzed and the top 100 molecules, which had a PLANTS [25] score at least 80% of the best PLANTS [25] score, were retrieved for further studies.

The further submission of those 100 molecules to MVD^®^ [22] and analysis of poses of ligands that interacted simultaneously with at least one catalytic residue (Glu177 and/or Arg180) [12] and one residue of the secondary site (Asp75, Asn78, Asp96, and/or Asp100) [13], resulted in 29 ligands whose best poses met those criteria. After the selection of the best pose per ligand according to criteria listed in Table 1, the ligands were divided into five groups, being clustered according to their structural characteristics (Figure S1).

**Figure 3:**
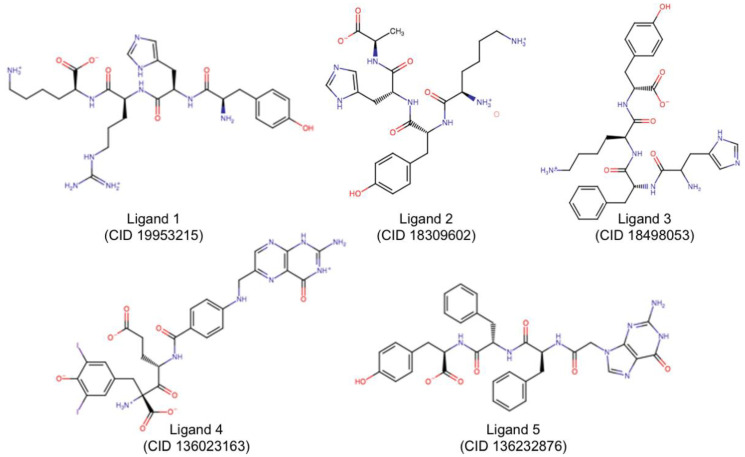
Molecules submitted to MD simulations.

In each group, Table 1 criteria were applied one more time to rank the selected pose of each ligand and then compare docking performances of different ligands (Table S2). The best ranked ligand of each group was then selected for further MD simulations (Table 2 and Figure 3). In Table 2, catalytic residues are marked in blue; residues of the secondary site are in green and other residues involved in substrate complexation are in yellow. Residues with no color filling are not known to participate in RTA catalytic activity.

In Figure 3, compound identifier (CID) numbers correspond to the PubChem (https://pubchem.ncbi.nlm.nih.gov/) identification of each molecule; and the number of the ligand is the same number of the group they belong to (i.e., Ligand 1 is the representative molecule of Group 1). All molecules are already in the protonation state previously defined by LigPrep [26]. NNPT (Figure 2) was also submitted to MD simulations in order to provide reference parameters for comparison.

### 2.4. Drug-Likeness Studies

All 29 molecules selected after docking studies using PLANTS [25] and MVD^®^ [22] programs were tested regarding their potential to be transformed in drugs. Although the results (Table 3) show that the ligands do not strictly follow all the rules, the drug-likeness results were not considered enough to eliminate any of those molecules at this stage of RTA inhibitors search since many approved drugs do not fall within the stablished ranges and have proven to be effective nevertheless, like Lipitor^TM^, Atorvastatin^TM^, and natural products [27,28]. Instead, those parameters can be useful to guide further improvements of the hits in this search. Additionally, the drug-likeness parameters are most suitable for drugs that are orally administered, which is not the mandatory uptake route for a ricin inhibitor. Actually, a ricin intoxication is an emergency situation that must be treated rapidly in order to minimize its effects on the human body, so a route of administration that provides a faster drug availability, such as an intravenous injection, would probably be a more suitable administration route for a ricin inhibitor then an oral uptake [29].

### 2.5. Molecular Dynamics Simulations

**Figure 4:**
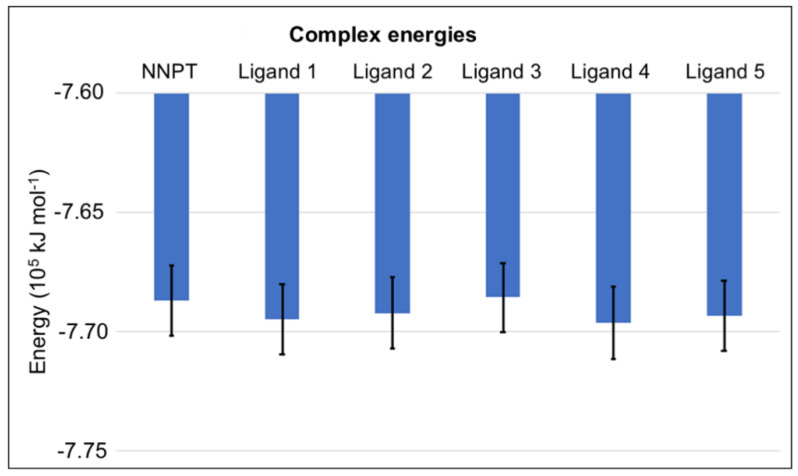
Mean and standard deviation values of energy during the MD simulations for the complexes RTA/Ligand.

Plots of total and mean energy of each protein-ligand complex over time (Figure S2) point to complex stabilization of all studied systems at the beginning of the MD simulation. Figure 4 shows a bar chart containing mean and standard deviation values of the complex energies. The low standard deviations and the similarity to the energy of the complex RTA/NNPT confirm stabilities of the complexes.

**Figure 5:**
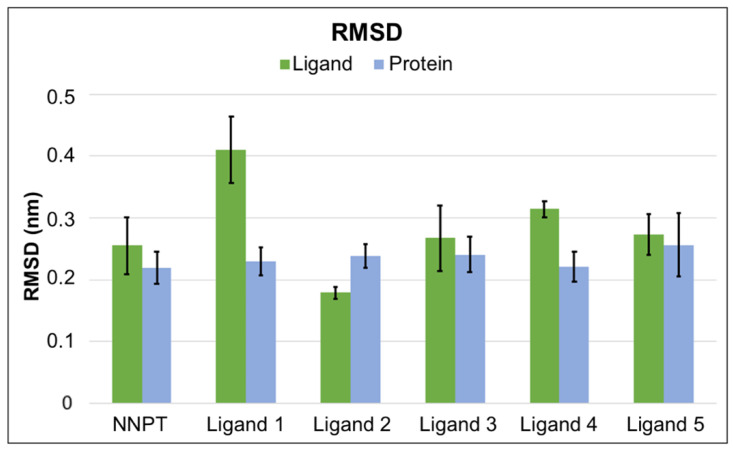
Mean and standard deviation of RMSD between 20 ns and 50 ns.

RMSD values were also analyzed to verify the stability of protein and ligand in each complex. A bar chart showing the mean and standard deviation of the RMSD values between 20 ns and 50 ns of simulation is shown in Figure 5.

Since the first 20 ns of MD simulation were considered to be enough for protein and ligand stabilization, the RMSD average and standard deviation values shown in Figure 5 are expected to be small. As can be seen, most systems behave well, showing low RMSD values; the exception was Ligand 1, which does not seem to stabilize so well in the protein pockets compared to the others. Ligand 2 behaved even better than the reference molecule, NNPT, showing an excellent stability inside RTA. Although Ligand 4 had a larger value of average RMSD, the standard deviation was quite low, also pointing to a good stabilization inside RTA. Ligands 3 and 5 behaved very similarly to NNPT; this suggests that, regarding the RMSD analysis, they may show similar performances to the reference molecule.

Root-mean-square fluctuations (RMSF) of RTA residues and radius of gyration of the protein over the simulation time were also analyzed and are shown in Figures S3 and S4, respectively. As expected, RMSF values indicate that the residues interacting with the protein fluctuate less and are, in most cases, the important residues listed in Table 1. The bar chart of the RTA radius of gyration during the MD simulations, in Figure S4, also points to protein stability once the low values observed indicate the protein compactness.

**Figure 6:**
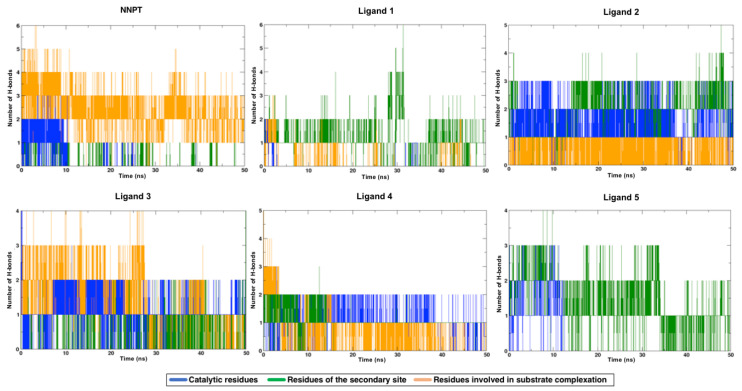
H-bonds between each ligand and RTA residues over time.

The analysis of H-bond interactions between each ligand and RTA was also conducted, and the results are shown in Figure 6. As can be seen, Ligands 2 and 3 are the most promising ones, since they interacted strongly with catalytic residues (shown in blue) and with secondary site residues (shown in green) during most of the simulation time, pointing to a good occupation of RTA both active and secondary sites. Although being structurally similar to Ligand 2 (Figure 3), Ligand 1 did not behave the same way and formed much less H-bonds with active site residues, which points to a lower potential of this ligand to inhibit RTA in experimental tests when compared to Ligand 2. Ligands 1 and 5 seem to be the least promising ones, since they interacted mostly with secondary site residues, leaving the active site free. Regarding Ligand 5, this is not unexpected since this ligand has a guanine ring, the very same nitrogenous base of rRNA that binds preferentially to the RTA secondary site. An analogous argument applies for Ligand 4, which showed little or no interactions with secondary site residues: this ligand has a pterin ring that is also present in the reference molecule NNPT (Figure 2) and is known to behave similarly to an adenine ring [14,15,16,30], the natural substrate of RTA inside the cell. Hence, although Ligand 4 appears to be long enough to bind residues of both pockets according to the docking studies, its high affinity for the active site contributes for the occupation of this site only and this is probably the reason why it interacted mainly with residues of the active site. The reference molecule, NNPT, formed H-bonds with catalytic residues until halfway of the MD simulation and, during the whole time, it interacted strongly with residues that are involved with substrate complexation (in orange in Figure 6). These results show that NNPT is more effective only than Ligand 1, to form and keep H-bonds with the key residues inside RTA. This reinforces our hypotheses that our ligands can bind more strongly and, therefore, be more efficient ricin inhibitors.

### 2.6. MM-PBSA Calculations

**Figure 7:**
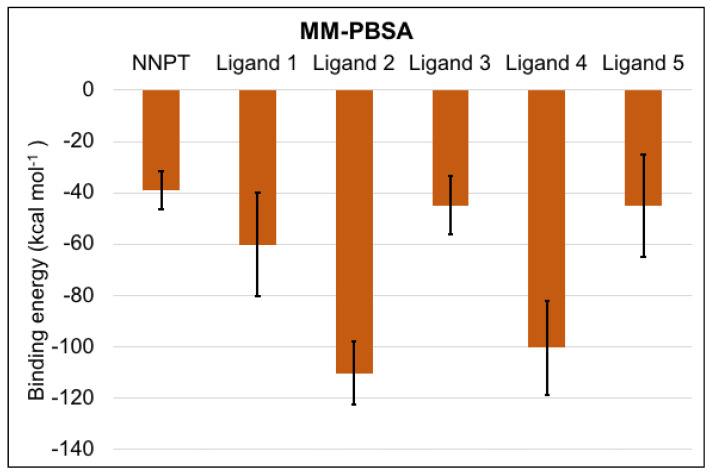
Mean and standard deviation values of the complexes binding energies calculated by the MM-PBSA method.

The values of mean and standard deviation of the binding energy between RTA and each ligand obtained through MM-PBSA calculations (Figure 7) are aligned with the previous docking and MD simulation results, since all ligands behave better than NNPT, showing more negative binding energies. Ligands 2 and 4 had the best results in terms of MM-PBSA binding energy, once again suggesting that these molecules are likely to be good hits in the discovery of RTA competitive inhibitors.

## 3. Discussion

Computational methods applied to toxicology have been proved, over the years, and through numerous works, as powerful methods in guiding the drug discovery of molecules capable of efficiently binding to biological targets, like proteins. These interactions can be exploited towards the discovery of antidotes to toxic proteins, drugs against pathogens, or modulators in the human body [19,31,32,33]. It is also possible to use cheminformatics aiming clarification and explanation of obtained or existing experimental results [15,34,35]. The application of these studies towards the development of toxin inhibitors has the additional advantage regarding safety, since they have the potential to diminish the need of dealing with toxic substances in early research stages. Our results are in line with this approach and suggest that the selected molecules have a high potential of showing inhibitory activity over RTA in experimental tests.

PLANTS [25] docking results showed that 100 molecules out of nearly 2500 had the top 80% best results, and this number of selected molecules lowered after MVD^®^ [22] docking analysis. After these two procedures, 29 molecules, representing approximately 1.1% of the initial set, met the defined criteria showed in Table 1. Although the number of selected molecules was small, their results were very promising in terms of potential to show RTA inhibitory activity. MVD^®^ [22] docking results shown in Table S2 showed several molecules with a lower MolDock score than the reference molecule, NNPT. This points to the formation of complexes with lower energy, and thus more stable, between those molecules and RTA. Additionally, all Ligands in Table 2 presented lower MolDock Scores than NNPT, so the representative molecule of each group is very promising to be an RTA inhibitor according to MVD^®^ [22] docking results.

MD simulations confirmed the docking studies since all tested molecules tended to stay bound to RTA. Once again, some molecules presented even better results than the reference molecule. Ligands 2, 3, and 4 (corresponding to PubChem (https://pubchem.ncbi.nlm.nih.gov/) CID 18309602, 18498053, and 136023163, respectively) were the ones with the best results in MD simulations and MM-PBSA calculations, indicating the potential of superior inhibition performances of these ligands in experimental tests when compared to NNPT, which is, currently, the compound holding the best experimental result against RTA in terms of IC_50_ values [15].

## 4. Conclusions

Computational techniques can be very useful in the discovery of new drug candidates, being an important technique to filter compound libraries and thus saving resources in experimental tests. In this work, an in silico approach was used aiming the identification of novel RTA inhibitors, in order to contribute to the process of drug discovery of new antidotes against ricin intoxication. Analysis of docking and MD simulations together with MM-PBSA calculations led to the selection of the three PubChem (https://pubchem.ncbi.nlm.nih.gov/) molecules CID 18309602, 18498053 and 136023163, as promising candidates for ricin antidotes, with the potential to be even more effective than NNPT (the current most effective in vitro inhibitor of RTA). Our theoretical results suggest that these three compounds worth being submitted to immediate experimental evaluation.

## 5. Materials and Methods

### 5.1. Protein Preparation and Redocking Procedure

The three-dimensional structure of RTA in complex with the inhibitor N-(N-(pterin-7-yl)carbonylglycyl)-L-phenylalanine was downloaded from Protein Data Bank (https://www.rcsb.org/) under the code 4HUO. Water molecules were removed of the structure prior to any docking or MD simulation and only RTA and the inhibitor remained for further computational work. Then, the cavity prediction tool of MVD^®^ [22] was used to detect the RTA cavities and to identify the active and the secondary sites of the protein, which was possible due to the knowledge about residues present in both pockets. The spherical search space was then defined, and its center and radius were chosen in a way that the search space could comprise both pockets.

In order to validate the docking procedure, the native ligand was submitted to redocking simulations in MVD^®^ [22], using the MolDock docking algorithm [22]. The best ranked pose according to the MolDock score was compared with the experimental position of this ligand in the PDB structure 4HUO through the RMSD value between these two positions.

### 5.2. Selection of the Reference Ligand, LBVS and Ligand Preparation, and Target Prediction

Our study started with the selection of the RTA competitive inhibitor with the lowest IC_50_ value found in the literature. The selected molecule was considered the reference structure and was given as input at PubChem database (https://pubchem.ncbi.nlm.nih.gov/) [17], where we performed a LBVS and selected all molecules that were at least 80% similar to the reference structure, according to the PubChem (https://pubchem.ncbi.nlm.nih.gov/) search method, which is based on the Tanimoto index [36,37]. All the selected molecules had their SMILES codes downloaded from PubChem website (https://pubchem.ncbi.nlm.nih.gov/), and those codes were submitted to LigPrep [26], where the three-dimensional structure of the molecules were generated using the Optimized Potentials for Liquid Simulations—all atoms (OPLS/AA) forcefield [38]. Additionally, LigPrep generated the protonated/deprotonated species of each molecule at pH 7.4 to simulate physiological conditions and estereoisomers where it was not previously defined.

Prior to docking simulations, the SMILES codes forming the library of ligands that are similar to the reference compound were submitted to HitPick web server (https://mips.helmholtz-muenchen.de/hitpick/cgi-bin/index.cgi?content=targetPrediction.html) in order to conduct an investigation regarding the predicted target of the aforementioned molecules. The predicted targets of the molecules were analyzed regarding the frequency that a specific target appeared and the precision of the algorithm to correctly predict the target of each molecule.

### 5.3. Molecular Docking

Two docking programs were used to refine results. Firstly, the PLANTS docking algorithm [25] available at Chemoinfo [24] was used because it is faster and a little less accurate, being suitable for early screenings of big sets of molecules. The inputs were the library formed after ligand preparation step, the protein structure with no ligands, and the center coordinates and radius of the search space. The best docking results were then retrieved by selecting the molecules that had PLANTS [25] score higher than 80% of the best PLANTS [25] score. Afterwards, those results were evaluated using MVD^®^ [22].

In MVD^®^ [22], 10 docking runs were carried out for each molecule selected in the previous step, with 10 poses returned per drug. All those poses were analyzed and the ones having positive MolDock score were excluded. Next, the poses were analyzed regarding the H-bonds they formed with RTA residues and poses that did not form H-bonds with at least one catalytic residue (Glu177, Arg180) and one residue from the secondary pocket (Asp75, Asn78, Asp96, Asp100) were also excluded. For each ligand, the remaining poses were ranked according to the criteria presented in Table 1. The best ranked pose of each ligand was then selected as the representative pose of that ligand.

Since the molecules were selected in PubChem (https://pubchem.ncbi.nlm.nih.gov/) by LBVS, many of them shared structural characteristics and were very similar to each other. Thereby, the ligands were separated into groups according to common features. In each group, the criteria of Table 1 were used again to rank the chosen pose of each ligand forming that group. The best ranked ligand of each group was selected for further MD simulations. NNPT, the reference molecule, was also submitted to docking simulations in MVD^®^ [22] in order to provide reference parameters for comparison.

### 5.4. Drug-Likeness Studies

All molecules selected after docking studies were analyzed regarding drug-likeness parameters using the software OSIRIS Property Explorer (https://www.organic-chemistry.org/prog/peo/). The parameters for drug-likeness evaluation were the molecule potential to cause mutagenic, tumorigenic, and/or irritant effects on the human body, as well as quantification of the calculated logarithm of partition coefficient (cLogP), solubility, molecular weight and number of hydrogen donors and acceptors in accordance with the Lipinski Rule of Five [39]. The reference values are shown in Table 4.

### 5.5. MD Simulations

MD simulations were carried out using GROMACS version 2019.4 [40]. Each molecule selected to MD simulations was first reparametrized using the software AnteChamber PYthon Parser interfacE (ACPYPE) [41] and MKTOP [42] in order to generate the topology and coordinate files that GROMACS with OPLS/AA forcefield [38] can recognize, since OPLS/AA itself has no parameters for the studied ligands. Topology and coordinate files of the protein were also generated using GROMACS with the same forcefield and then each ligand file was merged with the respective protein file in order to create coordinate and topology files for the protein–ligand system. The system was confined and centered in a dodecahedral box under periodic boundary conditions. The box type was chosen aiming calculation-time optimization, since the volume of a dodecahedral box is nearly 30% smaller than the volume of a cubic box with the same image distance, consequently reducing MD simulation time [43]. The minimum solute-box distance was set to 1.5 nm in order to guarantee a distance of at least 3.0 nm between the protein and its periodic image, avoiding artifacts during MD simulations and resulting in a box volume of 721 nm^3^. The box was filled with approximately 22,500 TIP4P water molecules [44] to reproduce solvent effects, and counterions were added to neutralize system charge.

MD simulations started with two 100-ps energy minimization (EM) steps conducted in sequence. The first EM was carried out with position restraint (PR) of protein and ligand so the water molecules could accommodate inside the box. Then, a second EM step was performed with no PR to reach a local minimum in the system potential energy surface. Both EM steps were conducted using steepest descent algorithm and the maximum force was set to 100.0 kJ mol^-1^ nm^-1^ as convergence criterion. In sequence, temperature and pressure equilibration were achieved by performing two 100-ps equilibration steps, first under an isothermal-isochoric (NVT) ensemble and, after, under an isothermal-isobaric (NPT) ensemble, to bring the system to 310 K temperature and 1 bar pressure. Both temperature and pressure were maintained using the Velocity-rescale thermostat [45] and Parrinello–Rahman pressure coupling methods [46], respectively. Finally, a 50 ns MD production step was conducted at 310 K and 1 bar using 2 fs as integration time, a cutoff of 1.2 nm for short-range (Lennard–Jones and Coulomb) interactions, and the leap-frog integrator algorithm; the coordinates of the complexes were stored every 10 ps. MD simulations were analyzed using the Visual Molecular Dynamics (VMD) [47] and Grace software [48].

### 5.6. MM-PBSA Calculations

The binding free energy of each protein–ligand complex submitted to MD simulations was estimated through MM-PBSA calculations in order to support the former results. The binding free energy was calculated by taking into account the *vacuum* potential energy, which includes both bonded and nonbonded interactions, as well as the free energy of solvation, which considers both polar and nonpolar terms. The polar solvation energy term is estimated by solving the Poisson–Boltzmann equation, and the nonpolar solvation energy term was calculated through the solvent accessible surface area (SASA) method [49,50,51,52]. MM-PBSA calculations were performed using the g_mmpbsa tool, compatible with the GROMACS software [53].

## Figures and Tables

**Table 1:** Pose ranking criteria.

Order	Criterion
First criterion	Largest number of catalytic residues interacting with the pose (Glu177 and Arg180)
Second criterion	Largest number of residues located in the secondary pocket interacting with the pose (Asp75, Asn78, Asp96, and Asp100)
Third criterion	Largest number of residues involved in substrate complexation interacting with the pose (Tyr80, Val81, Gly121, Tyr123, Asn209, Trp211)
Fourth criterion	Lowest MolDock score

**Table 2:** MVD^®^ [22] docking results of the poses selected for MD simulations.

Group	Molecule ^1^	MolDock Score (kcal mol^−1^)	Residues Forming H-bonds with the Pose ^3^
---	NNPT ^2^	−138.40	Arg180 Asn78 Tyr80 Val81
Group 1	19953215	−160.63	Glu177Arg180Asp75Asp96Asp100Tyr123Trp211 Asn122 Gly212 Arg258 Glu208
Group 2	18309602	−152.14	Glu177Arg180Asp75Asp96Asp100Tyr123Asn209 Asn122 Asp124 Glu208
Group 3	18498053	−161.20	Glu177Arg180Asn78Asp96Asp100Val81 Asn122 Ser176 Glu208 Arg258
Group 4	136023163	−203.93	Arg180Asn78Asp96Asp100Tyr80Val81Gly121Tyr123 Arg56 Thr77 Arg258
Group 5	136232876	−157.66	Arg180Asn78Asp96Asp100Trp211 Thr77 Asn122 Glu208 Gly212

^1^ For all molecules except NNPT, the number in this column corresponds to the PubChem compound identifier (CID). ^2^ NNPT docking results shown as reference. ^3^ Catalytic residues are lighted in blue; residues of the secondary site are in green, and other residues involved in substrate complexation are in yellow.

**Table 3:** Drug-likeness results.

Group	Molecule CID	Mut ^1^	Tumor	Irr	cLogP	Sol	Mol. Weight	Drug Score	H Donor	H Acceptor
---	NNPT	N	N	N	−2.16	−2.04	427	0.42	6	9
1	19953215	N	N	N	−6.85	−2.74	602	0.29	10	10
1	18305509	N	N	N	−6.85	−2.74	602	0.29	10	10
1	18493267	N	N	N	−6.85	−2.74	602	0.29	10	10
1	18243472	N	N	N	−5.52	−1.99	531	0.34	9	9
1	67312445	N	N	N	−5.52	−1.99	531	0.34	9	9
2	18309602	N	N	N	−5.77	−2.03	517	0.35	8	9
2	18309609	N	N	N	−6.13	−1.65	503	0.37	8	9
2	18499956	N	N	N	−6.13	−1.65	503	0.37	8	9
2	18305842	N	N	N	−7.46	−2.41	574	0.31	9	10
2	18500025	N	N	N	−5.77	−2.03	517	0.35	8	9
2	18306834	N	N	N	−7.46	−2.41	574	0.31	9	10
2	19953410	N	N	N	−6.13	−1.65	503	0.37	8	9
2	22659428	N	N	N	−6.99	−1.85	560	0.33	9	10
2	19953311	N	N	N	−5.20	−1.37	503	0.37	8	9
2	19953235	N	N	N	−6.99	−1.85	560	0.33	9	10
2	18309613	N	N	N	−7.46	−2.41	574	0.31	9	10
3	18498053	N	N	N	−4.33	−3.17	593	0.29	8	9
3	18500076	N	N	N	−4.33	−3.17	593	0.29	8	9
3	18500176	N	N	N	−4.67	−2.87	609	0.29	9	10
3	20044260	N	N	N	−4.33	−3.17	593	0.29	8	9
3	18492007	N	N	N	−3.00	−2.41	522	0.35	7	8
3	18500043	N	N	N	−4.67	−2.87	609	0.29	9	10
3	18499958	N	N	N	−3.00	−2.41	522	0.35	7	8
4	136023163	N	N	N	−2.59	−5.69	856	0.27	8	13
4	135977982	N	N	N	−3.36	−3.97	622	0.30	8	14
4	136149436	N	N	N	−4.02	−4.67	730	0.32	8	13
4	136132835	N	N	N	−2.92	−4.98	748	0.23	8	14
5	136232876	S	N	N	0.09	−4.35	666	0.14	7	9

^1^ Mut: mutagenic; Tumor: tumorigenic; Irr: irritant; Sol: solubility; Mol. Weight: molecular weight; H donor/acceptor: hydrogen donor/acceptor.

**Table 4:** Reference values for drug-likeness results.

	Mut ^1^	Tumor	Irr	cLogP	Sol	Mol. Weight	Drug Score	H Donor	H Acceptor
Reference values	N	N	N	<5	>−4	<500	Close to 1	<5	<10

^1^ Mut: mutagenic; Tumor: tumorigenic; Irr: irritant; Sol: solubility; Mol. Weight: molecular weight; H donor/acceptor: hydrogen donor/acceptor.

## Supplementary Materials

The following are available online at https://www.mdpi.com/2072-6651/12/12/746/s1, Table S1. Target prediction of the analyzed molecules; Figure S1. Groups of molecules formed after docking simulations and filtering; Table S2. MVD docking results of all selected molecules after docking simulations; Table S3. IUPAC names of the molecules of Table S2; Figure S2. Total and mean values of each complex energy; Figure S3. RMSF values of RTA residues when in complex with each ligand; Figure S4. Mean and standard deviation values of RTA radius of gyration when in complex with each ligand.
