# Supplementary material for: Ligand-Based Virtual Screening, Molecular Docking, Molecular Dynamics, and MM-PBSA Calculations towards the Identification of Potential Novel Ricin Inhibitors
Source: Toxins (Basel). 2020 Nov 26;12(12):746. doi: 10.3390/toxins12120746 (PMC7761309; doi:10.3390/toxins12120746)
Supplement: Supplementary file 1 [file toxins-12-00746-s001.pdf]

Supplementary Material: Ligand-Based Virtual Screening, Molecular Docking, Molecular Dynamics and MM-PBSA Calculations towards the Identification of Potential Novel Ricin Inhibitors

Fernanda D. Botelho, Marcelo C. dos Santos, Arlan da S. Gonçalves, Kamil Kuca, Martin Valis, Steven R. LaPlante, Tanos C. C. França and Joyce S. F. D. de Almeida

Table S1. Target prediction of the analyzed molecules.

Molecule[1]	Target[2]	Precision	Molecule[1]	Target[2]	Precision
46227250	PSMB5	96.7	18305749	SLC15A1	89.8
46227298	PSMB5	96.7	18305844	SLC15A1	89.8
50899573	PSMB5	96.7	18307628	SLC15A1	89.8
50899661	PSMB5	96.7	18309615	SLC15A1	89.8
74767133	PSMB5	96.7	18485316	AGTR2	89.8
90142755	PSMB5	96.7	18487924	AGTR2	89.8
145998685	PSMB5	94.8	18492868	SLC15A1	89.8
67890317	PSMB5	94.8	18493667	SLC15A1	89.8
67890575	PSMB5	94.8	18497039	SLC15A1	89.8
123701473	PSMB5	89.8	18497079	SLC15A1	89.8
135478902	DHFR	89.9	18497257	SLC15A1	89.8
135837087	FPGS	89.8	19950176	OPRD1	89.8
135974022	DHFR	89.8	19950183	OPRD1	89.8
136046101	FPGS	89.8	19950187	OPRD1	89.8
136087580	FPGS	89.8	19952950	OPRD1	89.8
136151232	DHFR	89.8	19952957	SLC15A1	89.8
136232994	OPRD1	89.8	19952969	OPRD1	89.8
136232996	OPRD1	89.8	19953184	OPRD1	89.8
145455884	SLC15A1	89.8	19953191	SLC15A1	89.8
145455908	SLC15A1	89.8	19953324	OPRD1	89.8
145455942	SLC15A1	89.8	19953326	OPRD1	89.8
145456012	SLC15A1	89.8	19953331	OPRD1	89.8
145456032	SLC15A1	89.8	19953342	OPRD1	89.8
145456171	SLC15A1	89.8	19953343	OPRD1	89.8
145457233	SLC15A1	89.8	19953450	OPRM1	89.8
145458655	OPRD1	89.8	19953570	OPRD1	89.8
145458662	SLC15A1	89.8	20005064	OPRM1	89.8
145458664	OPRD1	89.8	20008825	OPRM1	89.8
145458666	SLC15A1	89.8	20044256	OPRM1	89.8
145458671	OPRD1	89.8	20046632	OPRD1	89.8
145458943	LNPEP	89.8	22655308	PPYR1	89.8
18221409	SLC15A1	89.8	22655548	SLC15A1	89.8
18221446	SLC15A1	89.8	22655643	SLC15A1	89.8
18221486	SLC15A1	89.8	22657443	SLC15A1	89.8
18221578	SLC15A1	89.8	22659430	SLC15A1	89.8
18221602	SLC15A1	89.8	435685	SLC15A1	89.8

18221770	SLC15A1	89.8	44345945	OPRD1	89.8
18223224	SLC15A1	89.8	515713	SLC15A1	89.8
18231995	OPRD1	89.8	53372775	SLC15A1	89.8
18232002	SLC15A1	89.8	54286685	FPGS	89.8
18232004	OPRD1	89.8	67285433	PSMB5	89.8
18232007	SLC15A1	89.8	67285434	PSMB5	89.8
18232013	OPRD1	89.8	67285691	PSMB5	89.8
18232383	LNPEP	89.8	67285692	PSMB5	89.8
18235993	SLC15A1	89.8	67285886	PSMB5	89.8
18237118	SLC15A1	89.8	67286185	PSMB5	89.8
18241093	PPYR1	89.8	6992826	SLC15A1	89.8
18243731	PPYR1	89.8	92443036	SLC15A1	89.8
18243930	PPYR1	89.8	92443037	SLC15A1	89.8
18302890	PPYR1	89.8	92443038	SLC15A1	89.8
18305509	PPYR1	89.8	18305749	SLC15A1	89.8

[1] For all molecules, the number in this column corresponds to PubChem CID. [2] The targets are reported as gene symbols.

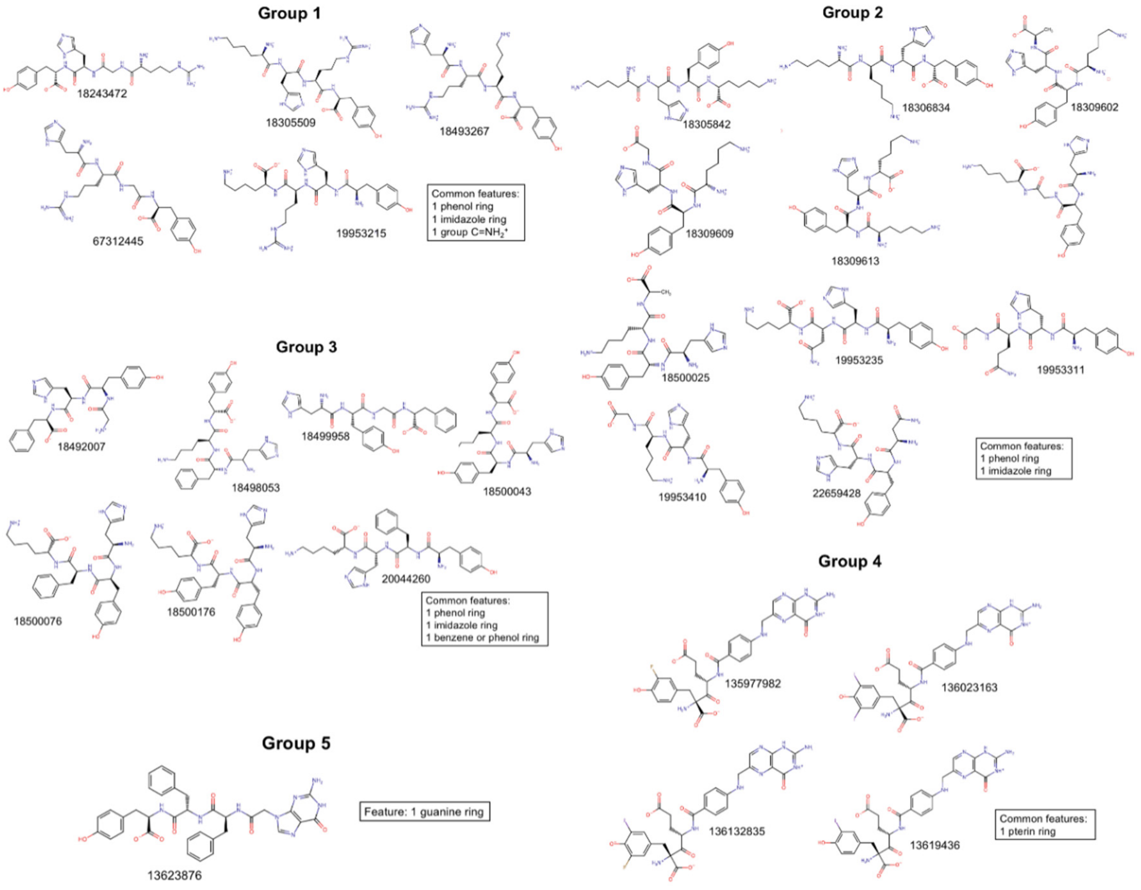

Figure S1. Groups of molecules formed after docking simulations and filtering.

Table S2. MVD docking results of all selected molecules after docking simulations.

Group	Molecule[1]	MolDock Score (kcal mol^{-1})	Residues Forming H-Bonds with the Pose[3]
---	NNPT[2]	−138.40	Arg180 Asn78 Tyr80 Val81
Group 1	19953215	−160,63	Glu177 Arg180 Asp75 Asp96 Asp100 Tyr123 Trp211 Asn122 Gly212 Arg258 Glu208
	18305509	−152.40	Glu177 Arg180 Asn78 Asp96 Asp100 Tyr80 Val81 Glu208 Ser176
	18493267	−126,73	Glu177 Arg180 Asp75 Asn78 Asp96 His94 Asn122 Glu208 Arg258
	18243472	−121.37	Glu177 Arg180 Asp75 Asp100 Glu208
	67312445	−128.75	Arg180 Asp75 Asn78 Asp96 Asp100 Arg134 Arg258
Group 2	18309602	−152.14	Glu177 Arg180 Asp75 Asp96 Asp100 Tyr123 Asn209 Asn122 Asp124 Glu208
	18309609	−144.04	Glu177 Arg180 Asn78 Asp96 Asp100 Val81 Tyr123 Glu208
	18499956	−137.87	Glu177 Arg180 Asn78 Asp96 Asp100 Val81 Thr77 Arg258
	18305842	−132.71	Glu177 Arg180 Asn78 Asp96 Asp100 Gly121 Val82 Phe93 Arg258
	18500025	−150.07	Glu177 Arg180 Asn78 Asp100 Val81 Gly121 Asn209 Asn122 Arg258
	18306834	−150.33	Glu177 Arg180 Asp96 Asp100 Gly121 Tyr123 Glu208 Gly212 Arg258
	19953410	−140.65	Glu177 Arg180 Asp96 Asp100 Val81 Trp211 Ser176 Arg258
	22659428	−173.78	Glu177 Arg180 Asn78 Asp96 Val81 His94 Glu208 Arg258
	19953311	−160.42	Glu177 Arg180 Asn78 Asp96 Asn209 Asn122 Glu208 Arg258
	19953235	−136.08	Glu177 Arg180 Asn78 Asp96 Arg48 Glu208
	18309613	−157.02	Arg180 Asp75 Asn78 Asp96 Asp100 Tyr80 Asn209 Asn122 Arg258
Group 3	18498053	−161.20	Glu177 Arg180 Asn78 Asp96 Asp100 Val81 Asn122 Ser176 Glu208 Arg258
	18500076	−164.04	Glu177 Arg180 Asp96 Asp100 Tyr80 Val81 Glu208 Gly212 Arg258
	18500176	−137.90	Glu177 Arg180 Asp96 Asp100 Val81 Gly121 Arg258
	20044260	−151.01	Glu177 Arg180 Asp75 Asn78 Tyr123 Arg56 Asn122 Glu208
	18492007	−140.43	Glu177 Arg180 Asn78 Asp96 Gly121
	18500043	−143.81	Glu177 Arg180 Asp96 Asp100 Asn122 Glu208 Gly212
	18499958	−129.06	Glu177 Arg180 Asn78 Val81 Gly121 Asn209 Thr77 Arg258

Group 4	136023163	−203.93	Arg180 Asn78 Asp96 Asp100 Tyr80 Val81 Gly121 Tyr123 Arg56 Thr77 Arg258
	135977982	−181.90	Arg180 Asn78 Asp96 Asp100 Val81 Gly121 Tyr123 Arg48 His94 Asn122
	136149436	−168.51	Arg180 Asn78 Asp96 Asp100 Val81 Gly121 Tyr123 Arg48 Arg56 Phe93
	136132835	−151.28	Arg180 Asn78 Asp100 Gly121 Tyr123 Arg48 Arg56 Ser176 Arg258
Group 5	136232876	−157.66	Arg180 Asn78 Asp96 Asp100 Trp211 Thr77 Asn122 Glu208 Gly212

[1] For all molecules except NNPT, the number in this column corresponds to PubChem CID. [2] NNPT docking results shown as reference. [3] Catalytic residues are highlighted in blue; secondary site residues are in green and residues involved in substrate complexation are in yellow. Residues with no color filling are not known to participate in RTA catalytic activity.

Table S3. IUPAC names of the molecules of Table S2.

Group	PubChem CID	IUPAC Name
Group 1	19953215	6-amino-2-[[2-[[2-[[2-amino-3-(4-hydroxyphenyl)propanoyl]amino]-3-(1H-imidazol-5-yl)propanoyl]amino]-5-(diaminomethylideneamino)pentanoyl]amino]hexanoic acid
	18305509	2-[[2-[[2-(2,6-diaminohexanoylamino)-3-(1H-imidazol-5-yl)propanoyl]amino]-5-(diaminomethylideneamino)pentanoyl]amino]-3-(4-hydroxyphenyl)propanoic acid
	18493267	2-[[6-amino-2-[[2-[[2-amino-3-(1H-imidazol-5-yl)propanoyl]amino]-5-(diaminomethylideneamino)pentanoyl]amino]hexanoyl]amino]-3-(4-hydroxyphenyl)propanoic acid
	18243472	2-[[2-[[2-[[2-amino-5-(diaminomethylideneamino)pentanoyl]amino]acetyl]amino]-3-(1H-imidazol-5-yl)propanoyl]amino]-3-(4-hydroxyphenyl)propanoic acid
	67312445	(2S)-2-[[2-[[(2S)-2-[[(2S)-2-amino-3-(1H-imidazol-5-yl)propanoyl]amino]-5-(diaminomethylideneamino)pentanoyl]amino]acetyl]amino]-3-(4-hydroxyphenyl)propanoic acid
Group 2	18309602	2-[[2-[[2-(2,6-diaminohexanoylamino)-3-(4-hydroxyphenyl)propanoyl]amino]-3-(1H-imidazol-5-yl)propanoyl]amino]propanoic acid
	18309609	2-[[2-[[2-(2,6-diaminohexanoylamino)-3-(4-hydroxyphenyl)propanoyl]amino]-3-(1H-imidazol-5-yl)propanoyl]amino]acetic acid
	18499956	6-amino-2-[[2-[[2-[[2-amino-3-(1H-imidazol-5-yl)propanoyl]amino]-3-(4-hydroxyphenyl)propanoyl]amino]acetyl]amino]hexanoic acid
	18305842	6-amino-2-[[2-[[2-(2,6-diaminohexanoylamino)-3-(1H-imidazol-5-yl)propanoyl]amino]-3-(4-hydroxyphenyl)propanoyl]amino]hexanoic acid
	18500025	2-[[6-amino-2-[[2-[[2-amino-3-(1H-imidazol-5-yl)propanoyl]amino]-3-(4-hydroxyphenyl)propanoyl]amino]hexanoyl]amino]propanoic acid
	18306834	2-[[2-[[6-amino-2-(2,6-diaminohexanoylamino)hexanoyl]amino]-3-(1H-imidazol-5-yl)propanoyl]amino]-3-(4-hydroxyphenyl)propanoic acid
	19953410	2-[[6-amino-2-[[2-[[2-amino-3-(4-hydroxyphenyl)propanoyl]amino]-3-(1H-imidazol-5-yl)propanoyl]amino]hexanoyl]amino]acetic acid

	22659428	6-amino-2-[[2-[[2-[(2,4-diamino-4-oxobutanoyl)amino]-3-(4-hydroxyphenyl)propanoyl]amino]-3-(1H-imidazol-5-yl)propanoyl]amino]hexanoic acid
	19953311	2-[[5-amino-2-[[2-[[2-amino-3-(4-hydroxyphenyl)propanoyl]amino]-3-(1H-imidazol-5-yl)propanoyl]amino]-5-oxopentanoyl]amino]acetic acid
	19953235	6-amino-2-[[4-amino-2-[[2-[[2-amino-3-(4-hydroxyphenyl)propanoyl]amino]-3-(1H-imidazol-5-yl)propanoyl]amino]-4-oxobutanoyl]amino]hexanoic acid
	18309613	6-amino-2-[[2-[[2-(2,6-diaminohexanoylamino)-3-(4-hydroxyphenyl)propanoyl]amino]-3-(1H-imidazol-5-yl)propanoyl]amino]hexanoic acid
Group 3	18498053	2-[[6-amino-2-[[2-[[2-amino-3-(1H-imidazol-5-yl)propanoyl]amino]-3-phenylpropanoyl]amino]hexanoyl]amino]-3-(4-hydroxyphenyl)propanoic acid
	18500076	6-amino-2-[[2-[[2-[[2-amino-3-(1H-imidazol-5-yl)propanoyl]amino]-3-(4-hydroxyphenyl)propanoyl]amino]-3-phenylpropanoyl]amino]hexanoic acid
	18500176	6-amino-2-[[2-[[2-[[2-amino-3-(1H-imidazol-5-yl)propanoyl]amino]-3-(4-hydroxyphenyl)propanoyl]amino]-3-(4-hydroxyphenyl)propanoyl]amino]hexanoic acid
	20044260	6-amino-2-[[2-[[2-[[2-amino-3-(4-hydroxyphenyl)propanoyl]amino]-3-phenylpropanoyl]amino]-3-(1H-imidazol-5-yl)propanoyl]amino]hexanoic acid
	18492007	2-[[2-[[2-[(2-aminoacetyl)amino]-3-(4-hydroxyphenyl)propanoyl]amino]-3-(1H-imidazol-5-yl)propanoyl]amino]-3-phenylpropanoic acid
	18500043	2-[[6-amino-2-[[2-[[2-amino-3-(1H-imidazol-5-yl)propanoyl]amino]-3-(4-hydroxyphenyl)propanoyl]amino]hexanoyl]amino]-3-(4-hydroxyphenyl)propanoic acid
	18499958	2-[[2-[[2-[[2-amino-3-(1H-imidazol-5-yl)propanoyl]amino]-3-(4-hydroxyphenyl)propanoyl]amino]acetyl]amino]-3-phenylpropanoic acid
Group 4	136023163	(2R,4S)-2-amino-4-[[4-[(2-amino-4-oxo-3H-pteridin-6-yl)methylamino]benzoyl]amino]-2-[(4-hydroxy-3,5-diiodophenyl)methyl]-3-oxoheptanedioic acid
	135977982	(2R,4S)-2-amino-4-[[4-[(2-amino-4-oxo-3H-pteridin-6-yl)methylamino]benzoyl]amino]-2-[(3-fluoro-4-hydroxyphenyl)methyl]-3-oxoheptanedioic acid
	136149436	(2R,4S)-2-amino-4-[[4-[(2-amino-4-oxo-3H-pteridin-6-yl)methylamino]benzoyl]amino]-2-[(4-hydroxy-3-iodophenyl)methyl]-3-oxoheptanedioic acid
	136132835	(2R,4S)-2-amino-4-[[4-[(2-amino-4-oxo-3H-pteridin-6-yl)methylamino]benzoyl]amino]-2-[(3-fluoro-4-hydroxy-5-iodophenyl)methyl]-3-oxoheptanedioic acid
Group 5	136232876	2-[[2-[[2-[[2-(2-amino-6-oxo-1H-purin-9-yl)acetyl]amino]-3-phenylpropanoyl]amino]-3-phenylpropanoyl]amino]-3-(4-hydroxyphenyl)propanoic acid

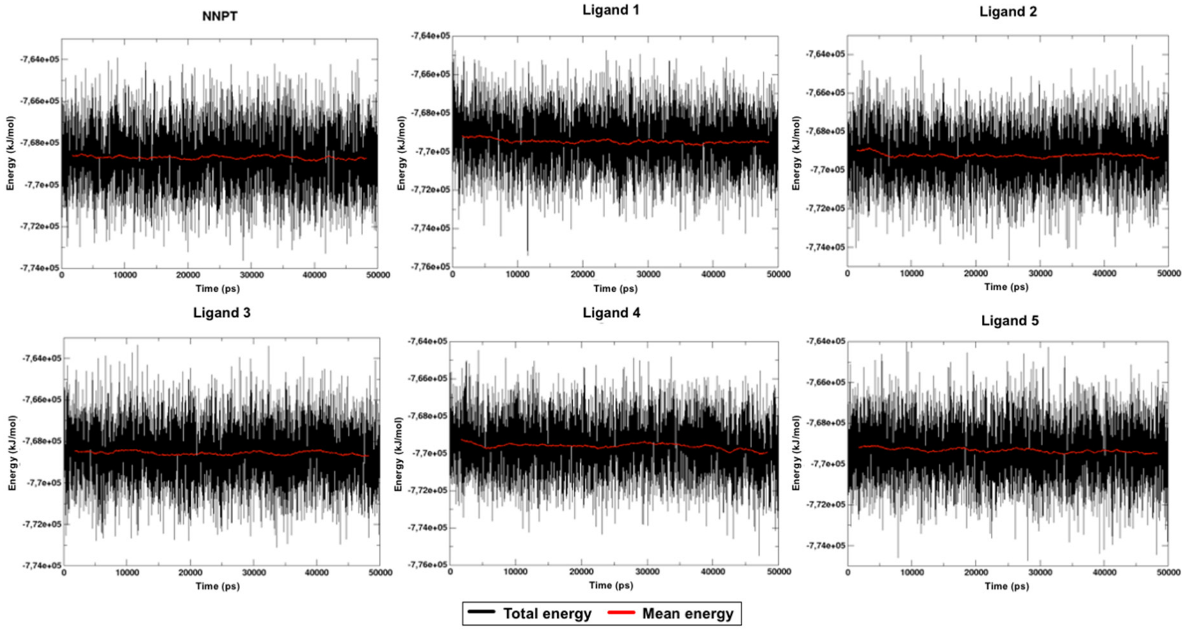

Figure S2. Total and mean values of each complex energy.

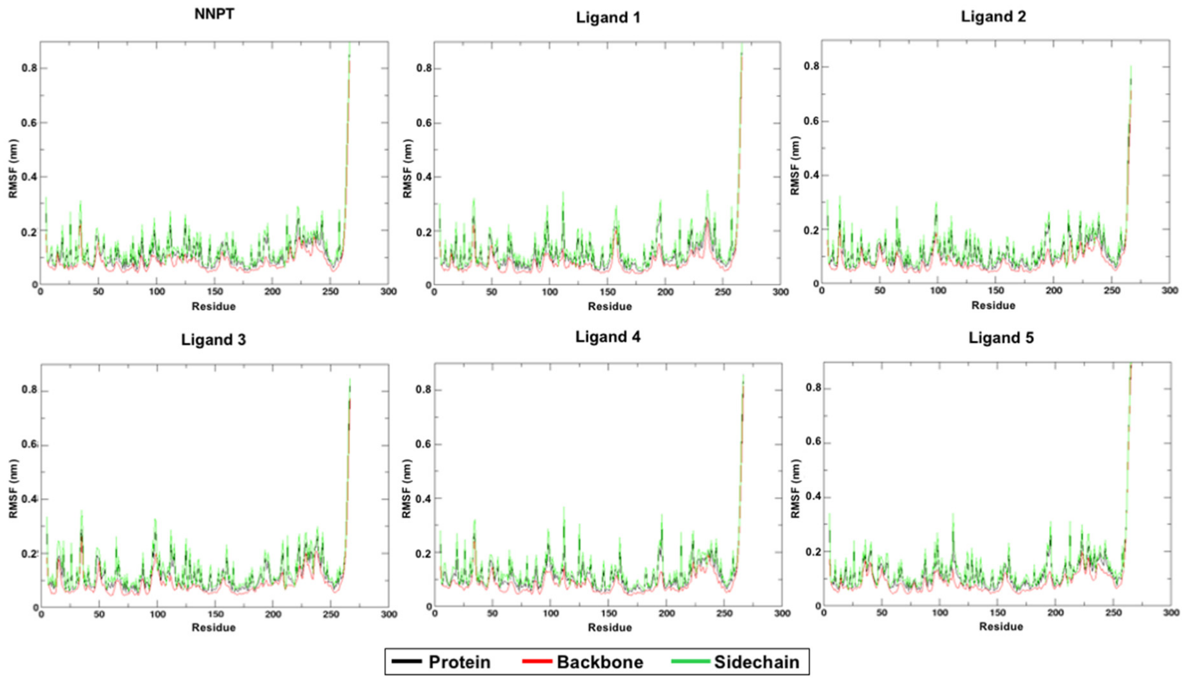

Figure S3. RMSF values of RTA residues when in complex with each ligand.

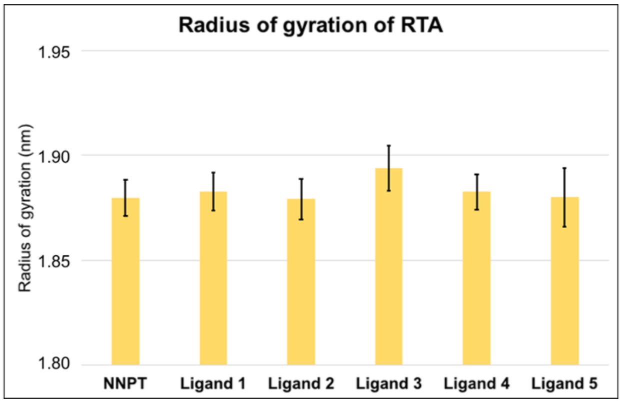

Figure S4. Mean and standard deviation values of RTA radius of gyration when in complex with each ligand.
